# A computational approach for identifying pseudogenes in the ENCODE regions

**DOI:** 10.1186/gb-2006-7-s1-s13

**Published:** 2006-08-07

**Authors:** Deyou Zheng, Mark B Gerstein

**Affiliations:** 1 Department of Molecular Biophysics and Biochemistry, Yale University, Whitney Avenue, New Haven, CT 06520, USA; 2 Department of Computer Science, Yale University, Prospect Street, New Haven, CT 06520, USA; 3 Program in Computational Biology and Bioinformatics, Yale University, New Haven, CT 06520, USA

## Abstract

**Background:**

Pseudogenes are inheritable genetic elements showing sequence similarity to functional genes but with deleterious mutations. We describe a computational pipeline for identifying them, which in contrast to previous work explicitly uses intron-exon structure in parent genes to classify pseudogenes. We require alignments between duplicated pseudogenes and their parents to span intron-exon junctions, and this can be used to distinguish between true duplicated and processed pseudogenes (with insertions).

**Results:**

Applying our approach to the ENCODE regions, we identify about 160 pseudogenes, 10% of which have clear 'intron-exon' structure and are thus likely generated from recent duplications.

**Conclusion:**

Detailed examination of our results and comparison of our annotation with the GENCODE reference annotation demonstrate that our computation pipeline provides a good balance between identifying all pseudogenes and delineating the precise structure of duplicated genes.

## Background

Pseudogenes occupy a significant portion of vertebrate genomes, and are especially prevalent in the mammalian genomes [1-6]. It is estimated that the human genome may contain approximately 20,000 pseudogenes and pseudogene fragments [1,4]. These are copies of functional genes that have lost their potential as DNA templates for functional products (for example, proteins). Usually, they have accumulated various detrimental sequence mutations (for example, nonsense mutation) during evolution. Based on the processes of their formations, pseudogenes are often separated into: processed pseudogenes, which have been retrotransposed back into a genome from mRNA intermediates; and non-processed pseudogenes [2,5,6].

Pseudogenes have traditionally been recognized as an important resource for exploring dynamics and evolutionary history of genes and genomes. The common wisdom is that pseudogenes are non-functional and evolve neutrally. Therefore, they are often used for calibrating the parameters in various models of molecular evolution. However, some pseudogenes are transcribed and a few of them have been indicated to be involved in biological processes [5,7-9]. While the functional roles of pseudogenes are yet to be elucidated with more studies, the prevalence of pseudogenes in mammalian genomes has been problematic for gene annotation [10,11]. Because of high sequence similarity with functional genes, pseudogenes can sometimes be mistakenly annotated as genes, especially in an automated annotation pipeline. The task of distinguishing real genes from duplicated pseudogenes (a subtype of non-processed) is even more challenging. Therefore, the correct identification of pseudogenes is not only essential for subsequent pseudogene studies *per se *but also important for the overall accuracy of gene annotation [11].

Several computational algorithms have been described previously for annotating human pseudogenes [1,4,9,10,12-16]. All of them identify pseudogenes based on their two key sequence properties: similarity to genes and non-functionality. In practice, the former is often characterized by the sequence similarity between a pseudogene and its closest functioning gene relative (referred to as the 'parent gene') in the present-day genome. The latter is somewhat more elusive but is most commonly manifested by the occurrence of disablements (that is, premature stop codons, frameshifts and indels) in the 'putative coding region' of a pseudogene. Using such features as a pseudogene signature, Zhang *et al*. [1] identified approximately 8,000 processed pseudogenes in the human genome. The total number of human pseudogenes has been estimated to be between 10,000 and 20,000 according to this study and analyses from other groups [1,4,12,13].

Here we describe our pseudogene annotation for the ENCODE regions in the human genome. Our current computational pipeline contains various modifications and improvements from previous methods [1,9,16], with a new emphasis on delineating the precise structures of pseudogenes arising from recent gene duplication. Unlike their processed counterparts, duplicated pseudogenes arise from gene duplication and usually have intron-exon like structures inherited from their gene ancestors. This structure is also present in a unitary pseudogene, which has no functional relative in the same genome. The constituents of these structures may be called 'pseudo-introns' and 'pseudo-exons', terms that will be used in this paper. Previously, such 'introns' were inferred by aligning a pseudogene's nucleotide sequence to its parent gene's protein [1,4,13,14,16]. They were then used to distinguish duplicated from processed pseudogenes. As a result, processed pseudogenes with insertions (for example, transposons) could be incorrectly classified as duplicated unless extra care had been taken [16]. Our current method examines the preservation of a parent gene's intron-exon structure in a pseudogene and uses it as direct evidence for identifying duplicated pseudogenes. Applying this approach to the ENCODE regions found 164 pseudogenes (note that this number refers to the status in August 2005), which overlap very well with a reference set of manually curated pseudogenes from the GENCODE research group [17]. In addition, we found that 16 duplicated pseudogenes have their 'introns' and 'exons' arranged in the same patterns as those of their parent genes. These results demonstrate that our pipeline can identify pseudogenes correctly, and, as importantly, can delineate the precise structures of duplicated pseudogenes.

## Results

### Overview of our pipeline and number of pseudogenes in ENCODE regions

Gene prediction usually starts with the building of gene models from a specific training set of genes [11]. These models are subsequently applied to predict genes in un-annotated genomic sequences. Many algorithms are presented in accompanying papers in this special ENCODE Genome Annotation Assessment Project (EGASP) issue [18]. The special characteristics of pseudogenes, on the other hand, have led researchers to adopt rather different strategies for their prediction. A homology-based approach like ours scans a genome for DNA sequences similar to a set of query genes. The resulting gene-like sequences are then scrutinized and those possessing pseudogene features are extracted. It is obvious that such a method requires a good set of known genes that is as complete and accurate as possible. After evaluating several data sources (data not shown), we decided to use the annotation from the ENSEMBL [19]. To be precise, we used version 29.35e (released in March 2005), which contained 24,194 genes (including 1,978 pseudogenes) and 28,479 proteins (composed of 292,306 non-redundant exons).

**Figure 1:**
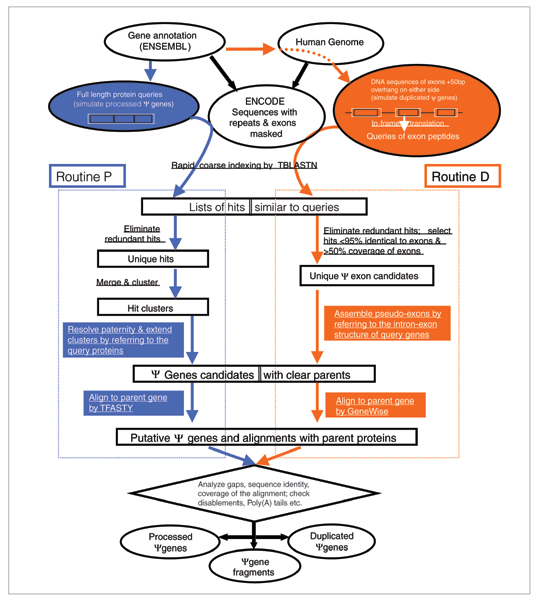
A flow chart of our computational pipeline for identifying pseudogenes. It contains two parallel procedures, one on the left (routine P) is mainly for processed pseudogenes and the other on the right (routine D) is for duplicated pseudogenes. The steps common to both are shown at the top and in the bottom. Both procedures searched the ENCODE regions for DNA sequences similar to human genes as annotated by the ENSEMBL. The two routines differ in how to perform the search and how to process the search results. The key differences are highlighted with blue in P and orange in D. At the end, an alignment between a known gene and a pseudogene candidate was constructed either by TFASTY or GeneWise. Information in this alignment and the computational path taken by a pseudogene were used together to separate pseudogenes into three classes: duplicated, processed and fragment.

One criterion commonly used for separating processed and non-processed pseudogenes is based on the occurrence of pseudo-intron(s). Processed pseudogenes should have no pseudo-introns as they are the consequence of retrotrans-position, but the non-processed ones typically retain introns or at least parts of them. In order to explore such a difference, we implemented a computational pipeline composed of two routines, with one (routine P) focusing on processed pseudogenes and the other (routine D) on duplicated ones (Figure 1). The major difference of these two routines lies in: the homology search step, where D uses individual exons while P uses full length proteins as queries; the step of assembling BLAST [20] hits into putative pseudogenes, where only D explicitly uses the intron-exon information of query genes (see Materials and methods for details). Putative pseudogenes from the P and D routines were combined and further inspected before they were finally classified. In our work, we specifically separated non-processed pseudogenes further into duplicated pseudogenes and fragments. The former have recognizable 'intron-exon' arrangements nearly identical to that of their parent genes whereas the latter do not.

In the end, the above pipeline identified a total of 211 pseudogenes (provided to EGASP/2005 in May 2005) in the ENCODE regions. Of these, 27 turned out to be LINE/SINE fragments after cross-reference with an updated version of RepeatMasker library. Excluding them, we identified 184 pseudogenes (Table 1), of which 93 were classified as processed, 19 as duplicated and 72 as pseudogene fragments. We also found one instance of a partially processed pseudogene; it is located at ENm011:80704-81919. The parent gene β-actin contains five exons. The processed pseudogene retains only the third intron (95 base-pairs (bp)) while the remaining three introns have been spliced out.

**Figure 2:**
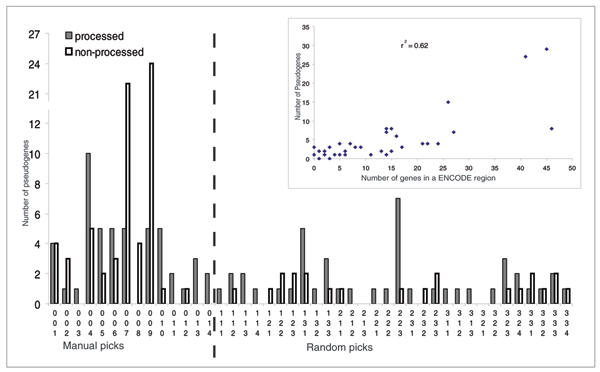
Distribution of 184 pseudogenes in ENCODE regions. Pseudogenes were first grouped into processed and non-processed (duplicated and fragments). Their numbers in the 44 ENCODE regions are plotted. The inserted panel shows that the number of pseudogenes is approximately correlated to that of genes within individual regions.

The 44 ENCODE regions were picked with a variety of gene densities and conservation [21]. As shown in Figure 2, the number of pseudogenes varies in different regions. Many have only one or two pseudogenes, but two (ENm007 and ENm009) contain more than 20. Both of these two regions are also gene dense [17]. ENm009 contains the well characterized β-globin locus and is known to have many olfactory receptor (OR) pseudogenes [22]. In fact, 24 of our 29 pseudogenes in ENm009 were identified with olfactory receptor genes as their parent genes. Since the coding region of an OR gene is intronless, all but one OR pseudogene were put into the group of pseudogene fragments in our pipeline. Overall, the number of pseudogenes appears to correlate well with the number of genes in individual regions (r^2 ^= 0.65) (Figure 2).

### Duplicated pseudogenes

Duplicated pseudogenes are an important evolutionary residue of a genome's past activity. It is generally thought that gene duplication is one of the main driving forces for creating genes with novel functions [23]. Therefore, the accurate identification of duplicated pseudogenes is valuable both for understanding the process of gene duplication and for studying the subsequent evolutionary fate of duplicated genes, which can either lead to gene death (that is, becoming a pseudogene or deleting a gene) or gene birth (that is, arising of a gene with new function). Only 19 of the 91 non-processed pseudogenes retain clear evidence of duplication, as supported by the preservation of intron-exon structures matching to their parent genes. The longest one contains 10 pseudo-exons spanning about 19,000 nucleotides. However, most of them (16) have lost at least one exon from their ancestors based on comparisons with their modern gene relatives. Notably, five of our duplicated pseudogenes are on the same chromosome as their parent genes, suggesting that they may have arisen from local gene duplication.

The majority of non-processed pseudogenes did not contain a pseudo-intron and, therefore, were classified as fragments. With the exception of OR pseudogenes, which originate from single-exon genes, most of them only match a short fragment of their parent proteins. They may represent single-exon duplication of their parent genes or have entirely lost their original intron-exon signatures. In this sense, it is appropriate to say that the duplicated pseudogenes identified by us arise from recent events of gene duplication.

### Brief comparison of data from routines P and D

We merged pseudogenes from our two computational routines. We have examined how pseudogenes were identified and labeled by these two routines. As shown in Table 1, nearly all processed pseudogenes were detected (and labeled correctly; data not shown) in routine P. Routine D is intended for duplicated pseudogenes, but we allow it to pick up processed ones as well (see Materials and methods for details). In fact, it recognized one-third of our final 93 processed pseudogenes with an additional three not detected in routine P. These three were quite short and shared rather weak sequence similarity with their parent genes, so they were filtered out in routine P.

As mentioned above, approximately 80% of non-processed pseudogenes did not have a pseudo-intron and in many cases could be reliably aligned to only a fraction (<70%) of their parent genes. These were classified as pseudogene fragments [1,16]. Since they did not contain detectable pseudo-introns, they look like 'processed pseudogenes' and were mainly identified from routine P as expected. Most (25) OR pseudogenes were in this class; they actually result from gene duplication but were classified as pseudogene fragments in our computational scheme.

Most final duplicated pseudogenes were discovered by both D and P routines. It might appear that this defeats the whole purpose of routine D. However, detecting the presence of a pseudogene is one thing but recovering its full structure with accurate pseudo-intron-exon boundaries is another. The goal of routine D is really the latter. For six cases, only part of the pseudogene was identified in routine P while the entire structure with pseudo-exons and pseudo-introns was correctly annotated in routine D. Furthermore, two of these six were labeled as processed in routine P. These inaccuracies would not have been corrected without information from routine D. On the other hand, to our surprise, three duplicated pseudogenes were missed in routine D. Further manual inspection showed that one in ENm001 (1092641-1094417) was more likely to be a processed pseudogene disrupted by a 1.2 kb DNA insertion; the other two (ENm006: 796815-805109; ENm008: 4095-8064) were almost identical (>95% sequence identity) to their parent genes. In routine D, we did not analyze genomic sequences with the latter feature.

In conclusion, the above results indicate that by combining two routines our computation pipeline provides a good balance between detecting all pseudogenes and identifying the exact structure of duplicated pseudogenes.

### Comparison with GENCODE/HAVANA annotation

The GENCODE group and the HAVANA team have produced a high quality manual annotation for ENCODE regions, including 521 genes and 167 pseudogenes [17]. These served as the gold standard for evaluating other prediction methods in the EGASP/2005 workshop [18]. Table 2 summarizes the comparison between our pseudogenes and HAVANA annotation; 136 of our 184 pseudogenes overlapped with 135 of their pseudogenes. One-quarter of pseudogenes was unique to each method. This is a very promising result since pseudogenes annotated by different methods often did not agree very well [9,14].

In addition, 95 and 20 of our pseudogenes intersected with introns and exons from GENCODE, respectively. The overlapping between our pseudogenes and exons raises an important issue. Our method uses annotated genes for two purposes: as queries to search for similar genomic sequences; and as filters to eliminate exon sequences (that is, remove known genes). In our work, the gene annotation was obtained from the ENSEMBL, which contained 576 predicted genes in the ENCODE regions. Any discrepancy between ENSEMBL and the HAVANA/GENCODE annotation would be carried over to our annotation. For example, six GENCODE pseudogenes overlapped with ENSEMBL exons and thus could not be found by us. Conversely, some pseudogenes in our list could be components of genes missed by the ENSEMBL. Of the 20 overlapping with GENCODE exons, 11 were classified as pseudogene fragments, suggesting that they probably are real exons missed in our gene collection.

**Figure 3:**
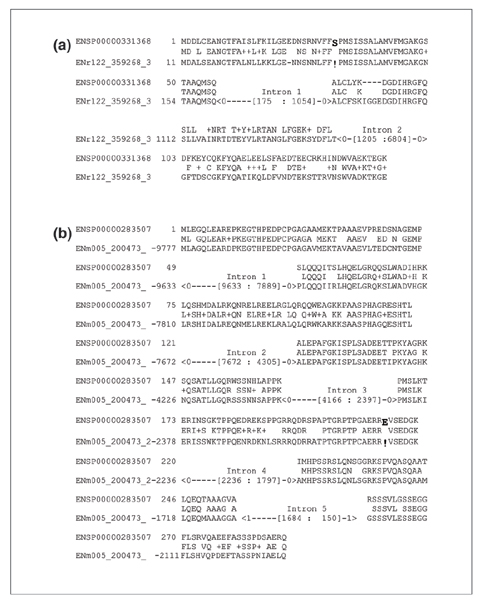
Two pseudogenes inconsistent with GENCODE gene annotation. **(a) **A pseudogene in ENr122: 359245-366200 (+) and its alignment with an ENSEMBL protein ENSP00000331368 (Serpin B8). This pseudogene overlaps a GENCODE gene whose transcript (ID: 'AC009802.2-001') contained the three pseudo-exons and one additional 5' exon. **(b) **A pseudogene at ENm005:200473-211501 (-) and its alignment with an ENSEMBL protein ENSP00000283507 (TCP-10 homolog). The first four pseudo-exons were included in a five-exon GENCODE transcript (ID: 'AP000274.7-001'). The frameshift mutations ('!' in the alignment) in both pseudogenes are highlighted.

In order to illustrate the difficulty of gene/pseudogene annotation, we present two cases of discrepancy between our pseudogene annotation and GENCODE's gene prediction. First, in ENr122, we predicted a duplicated pseudogene at 359245-366200. There is a frame shift mutation in this pseudogene as shown in its alignment with an ENSEMBL protein ENSP00000331368 (Figure 3a). The parent gene (Serpin B8 or CAP-2) is in very close vicinity at ENr122:375942-395286 (chr18: 59788243-59807587). This pseudogene contains three pseudo-exons corresponding to the first three exons of the parent gene, which has seven exons. It overlaps a GENCODE gene whose transcript (ID: 'AC009802.2-001') contains our three pseudo-exons and one extra untranslated exon at the 5' end. The disablement is in the first pseudo-exon (Figure 3a). However, if this disablement is skipped and an internal ATG is used as an alternative translation start site, a truncated protein can be produced. Without further experiments, the contradictory annotations for this region can not be resolved convincingly.

In the second case, we predicted a pseudogene at ENm005:200473-211501. Again, a frameshift mutation was found in the fourth pseudo-exon as shown in its alignment with an ENSEMBL protein ENSP00000283507 (Figure 3b). The parent gene (TCP-10) is in a different chromosome at chr6: 167554536-167579329. The pseudogene retains the intact structure of its parent gene with six exons and five introns. The first four pseudo-exons were included in a five-exon GENCODE transcript (ID: 'AP000274.7-001'). There is a full length cDNA (H-Inv: HIT000014684) matching this transcript, suggesting that this is likely a gene instead of pseudogene. However, transcription alone cannot be used as exclusive evidence to disapprove a pseudogene annotation because some pseudogenes are transcribed [9].

### Comparison with known pseudogenes

We compared our annotation to a few available known pseudogenes in the ENCODE regions. We began with four duplicated pseudogenes. Previously, a transcribed β-globin pseudogene (HBBP1) [24] in ENm009 was discovered with a substitution mutation in the start codon, a nonsense mutation in codon 15 and frameshift mutations in the second and third exons. It was detected by us and GENCODE annotators, but both predicted a shorter version (Table 3). We did not observe sequence similarity for the 1 kb sequence at the 3' end. Two α-globin and one ζ-globin pseudogene have also been described in ENm008 [25] (Table 3). One of the α-globin pseudogenes was present in the GENCODE annotation and ours as well. The other was missed by both groups because of its low sequence similarity to the parent gene, α-globin. (Note, we did find part of it during a homology search, but we did not pursue it because of its short sequence and no disablement.) The ζ-globin pseudogene is nearly identical to the ζ-globin gene except a single nonsense mutation in codon 7 [25]. It was identified in routine P but this stop codon was not displayed by alignment tools (see Discussion). Since this disablement was not visible and the remaining material was very similar (>95%) to the gene, we (perhaps over-cautiously) treated the sequence as a gene and did not report it.

Finally, previous studies have annotated many OR pseudogenes in ENm009 [22]. Since these are single exon pseudogenes, they are relatively easy to identify with computational pipelines. We found the majority of them and two examples are listed in Table 3.

## Discussion

Genes, especially protein coding genes, have been and will remain the major focus of research on the genome. The launch of the ENCODE project, however, aims to identify all structural and functional elements in the human genome [21]. Pseudogenes are a major component of our genome and a few of them have been suggested to have functions. Nevertheless, pseudogene annotation is often considered as a side-project or by-product of gene annotation. However, most pseudogenes have traceable origins and sequence features distinct from genes, suggesting that computational strategies specific to pseudogene prediction are necessary.

In this paper, we describe our general algorithm for annotating pseudogenes. For the EGASP held in May 2005, we identified 184 pseudogenes, of which 136 overlap with the reference set of pseudogenes manually curated by the GENCODE team. About a quarter of the pseudogenes are unique to our own method. Although pseudogene prediction was not part of the official competition in EGASP [18], it was discussed extensively during the workshop. In addition, several research groups have subsequently been working together to obtain an accurate pseudogene annotation in the 44 ENCODE regions, and to improve methods that can be applied to the entire genome.

### Limitation of our methods and future improvement

#### Gene annotation is in flux, so is pseudogene identification

Eighteen pseudogenes unique to our method were found to intersect with exons predicted by GENCODE (Table 2). Although a few of these pseudogenes may be bone *fide *pseudogenes, many of them are likely components of functional genes. A homology-based approach like ours needs gene annotation to compile a list of known genes (and proteins) as queries and as filters for eliminating genic sequences. Therefore, our result is limited by the source of gene annotation. Since annotation of the human genome is an ongoing dynamic process, our result will also be in flux. In this study, we used the ENSEMBL annotation [19] because of its good coverage of the human genome. It is also deeper than the RefSeq collection [26] but more specific than annotation derived purely from computation prediction using software like GenScan [11,27]. Having said that, we note that the ENSEMBL gene collection in itself includes some pseudogenes, due to the complexity of gene annotation as discussed above. For example, the human genome has three GAPDH and 80 ribosomal protein genes, but harbors approximately 80 GAPDH pseudogenes and approximately 1,700 ribosomal protein pseudogenes [1,13]. Some of these pseudogenes were incorrectly annotated as genes by ENSEMBL.

Another issue in relation to the quality of the data source is the correct identification of repetitive sequences. If these sequences are not masked, they could be easily annotated as pseudogenes simply because they have the features of pseudogenes (and they are pseudogenes in some sense). As a matter of fact, we mistook 27 LINE/SINE sequences as pseudogenes. It is fair to say we would have not annotated the above 20 gene components and 27 repeats as pseudo-genes if the relevant information was available to us in the beginning.

#### Need a better way to align a pseudogene to its parent protein sequence

The assessment of a genomic sequence as a pseudogene depends on the correct identification of its parent gene and the alignment between them. Currently, we assume that the most similar gene in the present-day genome represents the parent. This assumption may introduce unexpected artifacts into the alignment between a gene and its pseudogene relative, as both are descendents of an ancestral functional gene. Another practical issue is how to construct a 'correct' alignment. Stop codons and frameshifts are accommodated by programs like GeneWise [28] and TFASTY [29], but such disablements can break an alignment and leave it incomplete. Fundamentally, these programs are developed for genes so disablements are strongly disfavored in constructing alignments. This is the reason for our failure to identify the ζ-globin pseudogene in ENm008. This pseudogene contains a nonsense mutation in codon 7, but the rest of the sequence is identical to ζ-globin gene. Both GeneWise and TFASTY constructed an alignment starting from codon 8 that totally ignored the first seven codons. As a result, we overlooked this pseudogene. This case clearly indicates that a better tool is needed to align a pseudogene to its parent gene. An algorithm specifically designed for aligning pseudogenes [30] appears promising and a new program, GeneMapper [31], may be useful for addressing this problem.

#### Strength and limitation of our computational pipeline

Processed pseudogenes are derived from processed mRNA. They are usually not disrupted by large indels and thus easier to be identified than duplicated pseudogenes. Our pipeline, especially through routine P, is very good at identifying these pseudogenes. In routine P, the presence of pseudo-intron is inferred if an insertion (relative to its parent protein in the alignment) larger than a threshold (for example, 60 bp) is found in a pseudogene. As shown in Table 2 and discussed above, this parameter is sufficient for detecting most duplicated pseudogenes even though it may not lead to the identification of the full length pseudogenes. However, it will misclassify disrupted processed pseudogenes as duplicated ones. To overcome this limitation, we developed routine D, which explicitly uses the intron-exon structure of a parent gene to classify duplicated pseudogenes. This idea appears very reasonable but it assumes that the intron-exon structure of a gene is at least partially preserved in its pseudogene relatives. Further investigation will be required to validate this assumption. Nevertheless, the combination of routines P and D provides a good balance between discovering all pseudogenes and identifying the exact structure of duplicated pseudogenes.

## Conclusion

Using a homology-based approach, we have identified 184 pseudogenes in the ENCODE regions. The majority of them (74%) overlap with high quality pseudogenes annotated by the GENCODE group and the HAVANA team, an indication that our method worked successfully. Excluding the 20 pseudogenes overlapping with GENCODE exons, we would obtain a set of 164 pseudogenes, of which 91 are processed, 16 are duplicated, and the rest are tentatively classified as fragments. The list of our final 164 pseudogenes and the two ambiguous cases described above (Figure 3) can be found at [32]. This work also provides some insights for improving our approach in the future. At the current stage, there are not enough experimentally reported pseudogenes to establish a gold standard dataset for evaluating different prediction methods. However, several groups have worked together to reach a consensus and reliable list of pseudogenes for the ENCODE regions. Relevant information of that project is available at [33].

## Materials and methods

### Genomic sequence and annotated genes

The human genome sequence (build 35) was downloaded from the ENSEMBL [19] and sequences of the 44 ENCODE regions were extracted to serve as targets of our pseudogene annotation. Gene annotation was also obtained from the ENSEMBL. It included a set of known genes (as defined by ENSEMBL), with their intron, exon positions and their corresponding protein sequences.

Our computational pipeline contains two routines, with each focusing on a special type of pseudogene (Figure 1). In both routines, repetitive and exonic sequences in the ENCODE regions were masked.

### Routine P focuses on processed pseudogenes

Processed pseudogenes are generated by retrotransposition, the process of reverse transcription of a processed mRNA into DNA and its subsequent insertion into a genome. As a consequence, these pseudogenes usually do not contain pseudo-introns. Although some of them may contain indels, they generally can be reliably aligned to their parent proteins, and the alignments often expand the full parent sequences. In recognition of this, routine P (for 'processed') uses human protein sequences in their full lengths as queries to search for pseudogenes. The steps (Figure 1) involved in this routine have been described previously [1,16]. In brief, intergenic and intronic sequences similar (>40% sequence identity) to human proteins are identified. Putative pseudogenes covering >70% of their parent proteins without an insertion longer than 60 nucleotides are then labeled as processed pseudogenes, and those with a gap as duplicated. Pseudogenic sequences aligned to only part (<70%) of proteins are classified as pseudogene fragments.

### Routine D focuses on duplicated pseudogenes

A duplicated pseudogene usually contains pseudo-introns. To exploit this, we revised routine P and developed a new computational scheme, routine D (for 'duplicated'), which is more suitable for identifying duplicated pseudogenes (Figure 1). As described in detail below, there are two major changes from routine P: one during the homology search and the other in assembling search results into putative pseudogenes. In the homology search step, we used individual exons (of a gene) as our queries instead of the full length protein. When assembling pseudo-exons into pseudogene candidates, we referred to the intron-exon structures of our query genes to distinguish duplicated from processed pseudogenes.

#### Exon based BLAST

For each exon, we retrieved its DNA sequence and an extra 50 nucleotides adjacent to both ends of its exon (Figure 1). The extra 100 nucleotides are important for separating duplicated from processed pseudogenes because they enable our queries to span the pseudo-intron-exon boundaries of duplicated pseudogenes. All 'exonic' DNA queries were first in-frame translated to peptides and then used by the program TBLASTN [20] to search for similar sequences in the ENCODE regions.

#### Assemble BLAST hits

BLAST hits (that is, the sequence regions found by TBLASTN) were assembled into pseudogene candidates based on the intron-exon organization of their query genes. A hit (putative pseudo-exon) was skipped if it covered less than half of its query exon, or if it was nearly identical (>95% sequence identity) to its query. Based on their genomic coordinates, two neighboring hits were joined together and labeled as 'Dup' if: they were similar to adjacent exons of the same genes; and the distance between them was within the size (plus an extra 500 nucleotides) of the intron separating the two parent exons. They were otherwise labeled as 'NonDup' if they were separated by less than 50 nucleotides. This step noticeably considered the alignment running across the intron-exon boundary because the distance of two 'pseudo-exons' in a processed pseudogene would presumably be 0 or at least less than 50 nucleotides. This labeling step ran through all BLAST hits. In the end, neighboring hits were assembled into pseudogene candidates.

#### Identify the parent gene for a pseudogene

As expected, many pseudogenic regions shared sequence similarity to more than one gene. For such cases, we enforced a one-to-one relationship by picking the gene most similar (defined by the smallest e-value) to this region as its parent.

#### Align a pseudogene candidate to its parent protein

After the relationship between a pseudogene candidate and its parent was established, the pseudogenic DNA sequence and the gene's 'protein' sequence were retrieved from databases and then aligned using the program GeneWise [28]. We chose GeneWise because it allows frameshift mutations and can accommodate very large insertions. GeneMapper [31] will be an alternative to explore in the future. Furthermore, we also used information from the alignment to adjust the start and end positions of a pseudogene. From the final alignments, we defined the locations of pseudo-exons and pseudo-introns in reference to the parent proteins. Disablements in the aligned regions were also used as criteria for our pseudogene assignment. Finally, we compared a duplicated pseudogene to its parent gene's DNA sequence in order to validate our pseudogene classification and to refine the genomic locations of a duplicated pseudogene.

## Figures and Tables

**Table 1:** Separation of 184 pseudogenes in ENCODE regions identified in this study

Final pseudogene type*	Detected only by routine P	Detected by both routines	Detected only by routine D
Processed	60	30	3
Non-processed			
Duplicated	3	13^†^	3
Fragment	60	1	11

*The types are the final classification after information from routines P and D was combined. They could be different from a pseudogene's initial type labeled in either routine P or D. ^†^In routine P, two were annotated as processed and two as fragments and another four were identified partially.

**Table 2:** Overlapping of our 184 pseudogenes with GENCODE annotations

	GENCODE annotation			
	
Annotation in this study	Processed	Non- processed	Not annotated	Exons
Processed	70	7	13	3
Non-Processed	15	44	17	17
Not Annotated	15	18	-	-

**Table 3:** Examples of known pseudogenes in ENCODE regions

Name	Region	Pseudogene location	Our annotation	GENCODE annotation
β-globin	ENm009	488570-490726 (-)	489920-490351 (-)	488931-490348 (-)
α-globin	ENm008	156150-156704 (+)	NA	NA
α-globin	ENm008	158635-159503 (+)	158920-159084 (+)	158678-159333 (+)
ζ-globin	ENm008	152711-155400 (+)	NA	153121-155155 (+)
				
OR51H2P	ENm009	123369-124314 (+)	123353-124305 (+)	123368-124273 (+)
OR51B8P	ENm009	577399-578156 (-)	577369-578174 (-)	577403-578171 (-)

NA, not annotated.
